# Distributed Nonlocal Feedback Delays May Destabilize Fronts in Neural Fields, Distributed Transmission Delays Do Not

**DOI:** 10.1186/2190-8567-3-9

**Published:** 2013-07-30

**Authors:** Axel Hutt, Linghai Zhang

**Affiliations:** 1 INRIA Nancy, 615 rue du Jardin Botanique, 54600, Villers-lès-Nancy, France; 2 Department of Mathematics, Lehigh University, 14 East Packer Avenue, Bethlehem, PA, 18015, USA

**Keywords:** Traveling front, Spectral stability, Integro-differential equation, Distributed delay

## Abstract

The spread of activity in neural populations is a well-known phenomenon. To understand the propagation speed and the stability of stationary fronts in neural populations, the present work considers a neural field model that involves intracortical and cortico-cortical synaptic interactions. This includes distributions of axonal transmission speeds and nonlocal feedback delays as well as general classes of synaptic interactions. The work proves the spectral stability of standing and traveling fronts subject to general transmission speeds for large classes of spatial interactions and derives conditions for the front instabilities subjected to nonlocal feedback delays. Moreover, it turns out that the uniqueness of the stationary traveling fronts guarantees its exponential stability for vanishing feedback delay. Numerical simulations complement the analytical findings.

## 1 Introduction

The spatio-temporal dynamics of extended neuronal networks has attracted much attention in recent years [3,9,48,49]. They are powerful models to reproduce encephalographic data [40], to explain phenomena observed in medicine [39] such as general anaesthesia [26,29,34,44] and describe experimental spatio-temporal propagation of electric activity in neural tissues [23,38,41]. 

The spatially-extended neural network under study implies axonal connections with finite transmission speeds, which essentially leads to transmission delays. This delay depends strongly on the axonal branching architecture [2] and the degree of myelination of axonal branches [42]. For instance, unmyelinated axons exhibit a small transmission speed in the range of 0.1–1.0 m/s and occur mainly in short-range intracortical connections. In long-range axonal fibers such as cortico-cortical connections, the axons are myelinated leading to a faster transmission speed in the range of 1 m/s–100 m/s. Consequently, the resulting transmission delay between two spatial locations depend on the axonal paths and varies between 0.5 ms and 100 ms. Since these delay times are in the same range as time constants of synaptic responses of tens of milliseconds, effects of finite transmission delays on the spatio-temporal evolution of activity occur [6,30,46]. Although one may estimate such mean transmission delay times along axonal fibers, physiological studies point out that the transmission speed depends on the specific path the action potential takes, and hence varies in a single axonal branching structure from one neuron to another [42]. In addition, the detailed branching structure of neural tissue changes on a time scale of few months [45] or even few days [21], and hence changes the transmission speed. Consequently, it is not reasonable to study the effect of a single transmission speed, but rather a distribution of speeds. The present work considers such a distribution of transmission speeds and extends previous studies assuming a single speed only [13,30,37,38,46]. 

The model under study considers two types of axonal pathways. In intra-cortical connections, the lengths of axonal paths may vary due to the absence of fiber bundles of fixed length yielding a transmission delay proportional to the distance between two spatial locations. In contrast, cortico-cortical feedback connections may exhibit fiber bundles with fixed length yielding a constant feedback delay. By virtue of the distribution of transmission speeds, these two pathways are modeled by a distribution of transmission speeds and distributed feedback delays.

Most recent studies of extended neural networks considered either transmission speeds in intraarea connections or delay in feedback connections, although experimental findings indicate the presence of both connections [8], a distribution of transmission speeds [20] and feedback delays [8]. Only few previous studies consider both a single transmission delay in intraarea connections and the feedback delay [24,28,36,51]. The present work extends these studies by a detailed spectral stability analysis. 

It is well known that transmission delays and feedback delays may destabilize stationary activity yielding oscillatory phenomena [4,7,30]. To better understand the spatio-temporal dynamics of neural populations, it is essential to comprehend the role of delays and their effects on the activity propagation. The present work undertakes the analysis of stationary front for the general case of large classes of delay distributions and spatial interactions and, therefore, aims to reveal answers for this problem. 

## 2 Methods

This section introduces the model equation and gives the major previous results, which represent the basis of the novel results given in Sect. 3. To not expand the section too much, the discussion of the already published findings is kept short and concise.

### 2.1 The Model Equation

The model under study describes the activity in neural populations on a mesoscopic spatial and temporal scale with typical spatial range of 500 μm and temporal time constants of 5–10 ms. The corresponding spatial domain is coarse-grained and exhibits spatial patches. This structure reflects the macrocolumnar structure observed in primary sensory areas [19]. Such a neural field model allows the successful reproduction of electroencephalographic activity on the head [35] and the successful description of spiral waves in neural tissue [23]. More precisely, the neural field model describes a rate-coding neural population involving synaptic interactions whose spatio-temporal evolution obeys the following nonlinear scalar integro-differential equation:

(1) $$\begin{array}{rcl} \frac{\partial u}{\partial t}+u & = & \alpha \int_{0}^{\infty }\xi (c)\left[\int_{R}K(x-y)S\left(u\left(y,t-\frac{1}{c}|x-y|\right)-\theta \right)dy\right]dc\\ & & +\beta \int_{0}^{\infty }\eta (\tau )\left[\int_{R}W(x-y)S\left(u(y,t-\tau )-\theta \right)dy\right]d\tau ,\;t>0,\\ u(x,t) & = & u_{o}(x),\;-\infty <t\leq 0, \end{array}$$

where u=u(x,t) represents the mean membrane potential of a spatial patch at position *x* and time *t*[4] and $u_{o}$ is the initial activity. This model neglects units since they can be considered by appropriate scaling of time and space [27]. The prefactors α≥0 and β≥0 are nonnegative constants and reflect the synaptic weights of intraarea connections and feedback connections, respectively, and α+β>0. The function ξ≥0 represents the probability density distribution of axonal transmission speeds. Similarly, the function η≥0 represents the probability density function for feedback delays. We would like to point out that Eq. (1) describes the evolution of a minimal scalar model which, however, considers a large number of features. In spite of its reduced form, i.e., scalar and activity evolves in a one-dimensional space, and it promises to give insights into the effect of various delay types.

To learn more about the wave speed, at some point in the work, we will investigate the dependence of the wave speed of the traveling wave front on the transmission speeds and delays. To this end, the authors assume the axonal transmission speed distribution and the feedback delay distribution to a sum of two terms

(2) $$\xi (c)=\frac{1}{2}\left[\delta (c-c_{1})+\delta (c-c_{2})\right],\;\eta (\tau )=\frac{1}{2}\left[\delta (\tau -\tau_{1})+\delta (\tau -\tau_{2})\right],$$

i.e., the presence of two axonal transmission speeds and two feedback delays. This choice reflects the presence of short intracortical connections showing a small transmission speed [31] and small constant feedback delay and long-range cortico-cortical connections, which exhibit large axonal transmission speeds and large feedback delays. 

In addition, S=H(u−θ) denotes the transfer function of the model and is chosen to the Heaviside step function: H(u−θ)=0 for all u<θ, $H(0)=\frac{1}{2}$, and H(u−θ)=1 for all u>θ. This assumption is valid for identical neurons in the population [27]. Although this is a strong approximation, it gives first insights into the possible dynamics of homogeneous neural populations. The parameter *θ* is constant and represents the mean firing threshold of the neurons.

The spatial kernel functions *K* and *W* are real-valued and reflect two different nonlocal axonal connectivities between neurons and synapses: *K* represents intracortical axonal interaction in the neural population, which exhibits finite transmission speeds, whereas *W* denotes the axonal connectivity along fibers that leave the neural population and reenter it with a constant delay. These functions may be seen as weighted sums of probability density functions of axonal connections of subnetworks where the weights represent the synaptic strengths in each subnetwork [27]. We require that ξ=0 at least in a small open interval $\left(0,c_{0}\right)$, where $c_{0}>0$ is a positive constant. This assumption is reasonable since the resulting traveling wave front propagates with a wave speed $\mu_{0}$, $0<\mu_{0}<c_{0}$ that is equal to or smaller than the transmission speed $c_{0}$ due to physical reasoning. It is assumed that

(3) $$\begin{array}{rl} & 0<2\theta <\alpha +\beta ,\;\alpha K(0)+\beta W(0)>0,\\ & \int_{0}^{\infty }\xi (c)dc=1,\;\int_{0}^{\infty }\eta (\tau )d\tau =1,\\ & \int_{0}^{\infty }\frac{1}{c}\xi (c)dc<\infty ,\;\int_{0}^{\infty }e^{\tau }\eta (\tau )d\tau <\infty ,\\ & \left|K(x)\right|+\left|W(x)\right|\leq C\exp\left(-\rho |x|\right)\;\text{on }R,\\ & \int_{R}K(x)dx=1,\;\int_{R}W(x)dx=1,\\ & \int_{-\infty }^{0}K(x)dx=\frac{1}{2},\;\int_{-\infty }^{0}W(x)dx=\frac{1}{2},\\ & \int_{-\infty }^{0}|x|K(x)dx\geq 0,\;\int_{-\infty }^{0}|x|W(x)dx\geq 0,\\ & \int_{0}^{\infty }\left[\alpha K(x)+\beta W(x)\right]\exp\left(\frac{x}{c_{0}}\right)dx>\frac{\alpha +\beta }{2}-\theta \end{array}$$

for two positive constants C>0 and ρ>0.

To investigate various superpositions of subnetworks, the paper considers the following three general classes of synaptic interactions: 

(A) This class consists of all nonnegative kernel functions, reflecting global excitation in the neuronal population, i.e., K≥0 on ℝ.

(B) This class consists of all Mexican hat kernel functions, reflecting lateral inhibition and local excitation in the neuronal population, where each kernel function satisfies the conditions K≥0 on (−M,M) and K≤0 on (−∞,−M)∪(M,∞), for a positive constant M>0. This neural interaction is a successful model for interactions in visual receptive fields, e.g., to explain orientation tuning [5,47]. 

(C) This class consists of all upside down Mexican hat kernel functions, reflecting lateral excitation and local inhibition, where each kernel function satisfies the conditions K≤0 on (−M,M) and K≥0 on (−∞,−M)∪(M,∞), for a positive constant M>0. This interaction is motivated by the physiological finding [32] that networks of inhibitory neurons act locally only and excitatory neurons rarely exhibit local connections, but rather long-range interactions. 

 The synaptic feedback coupling *W* satisfies the same assumptions as *K* and it belongs to one of the three classes. Certainly, *K* and *W* are not necessarily in the same class. Moreover, *K* and *W* are not necessarily symmetric functions.

### 2.2 The Standing Wave Front

In the presence of a single firing threshold *θ* as in Eq. (1), a traveling wave front connects two constant states, i.e., one above and one below the threshold *θ*. Let β=0 and choose u(x,t)=u(t) in Eq. (1), then

(4) $$u^{\prime }(t)+u(t)=\alpha H\left(u(t)-\theta \right),$$

with two exponentially stable stationary states $U_{\mathrm{stationary}-0}=0$ and $U_{\mathrm{stationary}-1}=\alpha$. They are stable irrespective of the spatial interactions and the delay distributions.

Suppose that α+β=2θ and αK(0)+βW(0)>0. Now let us consider a stationary standing wave front with profile U=U(x). Without loss of generality, suppose that the front crosses the threshold *θ* at the point $x=x_{0}$, that is, U<θ on $\left(-\infty ,x_{0}\right)$, $U(x_{0})=\theta$, $U^{\prime }(x_{0})>0$ and U>θ on $\left(x_{0},\infty \right)$. Moreover, suppose that $\lim_{x\to -\infty }U(x)=0$, $\lim_{x\to \infty }U(x)=\alpha +\beta$ and $\lim_{x\to \pm \infty }U^{\prime }(x)=0$. Plugging such a solution back into the equation, noting that the solution is independent of time, we get

(5) $$\begin{array}{rcl} U(x) & = & \int_{R}\left[\alpha K(x-y)+\beta W(x-y)\right]H\left(U(y)-\theta \right)dy\\ & = & \int_{x_{0}}^{\infty }\left[\alpha K(x-y)+\beta W(x-y)\right]dy\\ & = & \int_{-\infty }^{x-x_{0}}\left[\alpha K(z)+\beta W(z)\right]dz, \end{array}$$

(6) $$U^{\prime }(x)=\alpha K(x-x_{0})+\beta W(x-x_{0}),$$

(7) $$U^{\prime }(x_{0})=\alpha K(0)+\beta W(0)>0.$$

Amari had studied solutions similar to (5) for general stationary states in his celebrated work [3]. Section 3.1 will elaborate in some detail the spectral stability of the stationary standing wave front given by (5).

It has been shown in many previous studies [4,27] that certain spatial interactions may destabilize spatially constant stationary states subject to the nonlinear gain function S=H(u−θ). In the present model, the functional derivative of the nonlinear gain function is given by δS[u(x,t)]/δu(x,t)=δ(u(x,t)−θ). In other words, spatial interactions at a certain spatial location *x* with activity u(x,t) contribute to the stability of a stationary state only if the location is close to the threshold *θ*.

### 2.3 The Traveling Wave Front and Its Stability

After the study of the existence of a standing wave front, this subsection focuses on the existence of a travelling wave front. Let α≥0, β≥0, θ>0 be constants such that 0<2θ<α+β. As shown in the previous section, there are two constant solutions $U_{1}=0$ and $U_{2}=\alpha +\beta$ to Eq. (1).

#### 2.3.1 The Front Shape

Suppose that u(x,t)=U(x+μt) is a traveling wave front of Eq. (1), where μ>0 represents the wave speed and z=x+μt represents a moving coordinate. Due to translation invariance, suppose that the traveling wave front satisfies the conditions U<θ on (−∞,0), U(0)=θ, $U^{\prime }(0)>0$ and U>θ on (0,∞). Suppose that the front satisfies the boundary conditions $\lim_{z\to -\infty }U(z)=0$, $\lim_{z\to \infty }U(z)=\alpha +\beta$ and $\lim_{z\to \pm \infty }U^{\prime }(z)=0$. For physical reasons, suppose that the wave speed satisfies the conditions $0<\mu <c_{0}$, where $c_{0}=\sup\{c>0:\xi =0\text{ on }(0,c)\text{ and}\;\xi \geq 0\text{ on }(c,\infty )\}$ is the smallest occurring transmission speed. Then the traveling wave front U=U(z) and the wave speed *μ* satisfy the equation

$$\begin{array}{rcl} \mu U^{\prime }+U & = & \alpha \int_{0}^{\infty }\xi (c)\left[\int_{R}K(z-y)H\left(U\left(y-\frac{\mu }{c}|z-y|\right)-\theta \right)dy\right]dc\\ & & +\beta \int_{0}^{\infty }\eta (\tau )\left[\int_{R}W(z-y)H\left(U(y-\mu \tau )-\theta \right)dy\right]d\tau . \end{array}$$

After a series of change of variables (such as $\omega =y-\frac{\mu }{c}|z-y|$ and $x=\frac{c}{c+s(z-\omega )\mu }(z-\omega )$, etc.), this above equation becomes

$$\begin{array}{rcl} \mu U^{\prime }+U & = & \alpha \int_{0}^{\infty }\xi (c)\left[\int_{-\infty }^{cz/(c+s(z)\mu )}K(x)dx\right]dc\\ & & +\beta \int_{0}^{\infty }\eta (\tau )\left[\int_{-\infty }^{z-\mu \tau }W(x)dx\right]d\tau , \end{array}$$

where s=s(x) is the sign function, which is defined by s(x)=−1 for all x<0, s(0)=0 and s(x)=1 for all x>0. By using the integrating factor idea and integration by parts, we find the representation of the front

(8) $$\begin{array}{rcl} U(z) & = & \alpha \int_{0}^{\infty }\xi (c)\left[\int_{-\infty }^{cz/(c+s(z)\mu )}K(x)dx\right]dc\\ & & -\alpha \int_{0}^{\infty }\xi (c)\left[\int_{-\infty }^{z}\exp\left(\frac{x-z}{\mu }\right)\frac{c}{c+s(x)\mu }K\left(\frac{cx}{c+s(x)\mu }\right)dx\right]dc\\ & & +\beta \int_{0}^{\infty }\eta (\tau )\left[\int_{-\infty }^{z-\mu \tau }W(x)dx\right]d\tau \\ & & -\beta \int_{0}^{\infty }\eta (\tau )e^{\tau }\left[\int_{-\infty }^{z-\mu \tau }\exp\left(\frac{x-z}{\mu }\right)W(x)dx\right]d\tau . \end{array}$$

The derivative of U=U(z) is given by

(9) $$\begin{array}{rcl} U^{\prime }(z) & = & \frac{\alpha }{\mu }\int_{0}^{\infty }\xi (c)\left[\int_{-\infty }^{z}\exp\left(\frac{x-z}{\mu }\right)\frac{c}{c+s(x)\mu }K\left(\frac{cx}{c+s(x)\mu }\right)dx\right]dc\\ & & +\frac{\beta }{\mu }\int_{0}^{\infty }\eta (\tau )e^{\tau }\left[\int_{-\infty }^{z-\mu \tau }\exp\left(\frac{x-z}{\mu }\right)W(x)dx\right]d\tau . \end{array}$$

These expressions are useful in a later part of the work.

#### 2.3.2 The Wave Speed of the Traveling Wave Front

To compute the wave speed, setting z=0 and U(0)=θ in (8) and making some simple change of variables yields

$$\begin{array}{rcl} \theta & = & \frac{\alpha +\beta }{2}-\alpha \int_{0}^{\infty }\xi (c)\left[\int_{-\infty }^{0}\exp\left(\frac{c-\mu }{c\mu }x\right)K(x)dx\right]dc\\ & & -\beta \int_{0}^{\infty }\eta (\tau )\left[\int_{-\mu \tau }^{0}W(x)dx\right]d\tau \\ & & -\beta \int_{0}^{\infty }\eta (\tau )e^{\tau }\left[\int_{-\infty }^{-\mu \tau }\exp\left(\frac{x}{\mu }\right)W(x)dx\right]d\tau . \end{array}$$

This equation may be rewritten as

(10) $$\phi (\mu )\equiv \phi_{1}(\mu )+\phi_{2}(\mu )=\frac{\alpha +\beta }{2}-\theta ,$$

where $\phi_{1}=\phi_{1}(\mu )$ and $\phi_{2}=\phi_{2}(\mu )$ are called speed index functions, defined on $\left(0,c_{0}\right)$, by

(11) $$\phi_{1}(\mu )=\alpha \int_{0}^{\infty }\xi (c)\left[\int_{-\infty }^{0}\exp\left(\frac{c-\mu }{c\mu }x\right)K(x)dx\right]dc,$$

(12) $$\begin{array}{rcl} \phi_{2}(\mu ) & = & \beta \int_{0}^{\infty }\eta (\tau )\left[\int_{-\mu \tau }^{0}W(x)dx\right]d\tau \\ & & +\beta \int_{0}^{\infty }\eta (\tau )e^{\tau }\left[\int_{-\infty }^{-\mu \tau }\exp\left(\frac{x}{\mu }\right)W(x)dx\right]d\tau . \end{array}$$

Define the sub speed index functions $\phi_{21}=\phi_{21}(\mu )$ and $\phi_{22}=\phi_{22}(\mu )$ on $\mu \in (0,c_{0})$ by

(13) $$\phi_{21}(\mu )=\beta \int_{0}^{\infty }\eta (\tau )\left[\int_{-\mu \tau }^{0}W(x)dx\right]d\tau ,$$

(14) $$\phi_{22}(\mu )=\beta \int_{0}^{\infty }\eta (\tau )e^{\tau }\left[\int_{-\infty }^{-\mu \tau }\exp\left(\frac{x}{\mu }\right)W(x)dx\right]d\tau .$$

There holds $\phi_{2}(\mu )=\phi_{21}(\mu )+\phi_{22}(\mu )$. We will see that there exists a unique positive solution (that is, the wave speed $\mu_{0}$) to the equation $\phi (\mu )=\frac{\alpha +\beta }{2}-\theta$.

Interestingly, the speed index functions $\phi_{1}$ and $\phi_{2}$ allow us to express the slope of the front at the threshold *θ* by utilizing (9) and (10)

(15) $$U^{\prime }(0)=\frac{1}{\mu_{0}}\left[\phi_{1}(\mu_{0})+\phi_{22}(\mu_{0})\right]=\frac{1}{\mu_{0}}\left[\frac{\alpha +\beta }{2}-\theta -\phi_{21}(\mu_{0})\right].$$

These expressions will be useful in later discussions of the spectral stability of the traveling wave front.

Moreover, for single transmission speed and single feedback delay (i.e., $\xi (c)=\delta (c-c_{0})$ and $\eta (\tau )=\delta (\tau -\tau_{0})$, where $c_{0}>0$ and $\tau_{0}>0$ are positive constants) and identical spatial kernel functions K(x)=W(x), we have

(16) $$\lim_{c_{0}\to \infty }\phi_{1}(\mu_{0})=\lim_{\tau_{0}\to 0}\phi_{2}(\mu_{0}).$$

Thus, there is no distinction between intracortical and feedback interactions. This is obvious from Eq. (1) where the two integrals may be written as a single one.

#### 2.3.3 The Spectral Stability of the Traveling Wave Front

Real neural structures exhibit a certain level of background noise, which may disturb the propagation of activity. Hence, it is important to study the stability of the stationary traveling wave front with respect to small perturbations. This kind of analysis has already been performed before for similar equations in [4,14,16,43]. 

Previous studies on integral and/or partial differential equations involving infinite delays have found criteria for the existence, uniqueness, and stability of traveling wave solutions [1,18]. Following the successful extrapolation approach for infinite delays in integral and/or partial differential equations based on finite delays [12,18] and recalling the close relationship of partial differential equations and integro-differential equations of the type discussed here [25], we assume in the following that mathematical analysis based on finite delays are applicable. An additional discussion of this approximation may appear necessary, but is neglected since it would exceed the major aim of the present work. Motivated by a previous study of Coombes and Owen [10], we study the spectral stability of the traveling wave front of (1) by constructing Evans functions which are well known from the literature of integral and/or partial differential equations [43]. 

To study the spectral stability of the front, we must rewrite the equation under consideration in moving coordinate and linearize the new equation with respect to the traveling wave front to obtain a linear equation. Then we seek solutions of the form exp(λt)ψ(z) to separate time and the moving coordinate *z* and to obtain an eigenvalue problem with the complex eigenvalue *λ* and the eigenfunction *ψ*; see also the Appendix. Solving the eigenvalue problem yields the definition of the Evans function. It turns out that a complex number $\lambda_{0}$ is an eigenvalue of the eigenvalue problem if and only if $\lambda_{0}$ is a zero of the Evans function. Note that $\lambda_{0}=0$ is always an eigenvalue, reflecting the translation invariance of the travelling wave front. Previous studies have shown that the equation E(λ)=0 determines the isolated spectrum $\left\{\lambda_{n}:n=1,2,3,\ldots \right\}$. As before, the stationary front is unstable if there exists some eigenvalue $\lambda_{0}$ with positive real part Reλ0>0 or if the neutral eigenvalue $\lambda_{0}=0$ is not simple.

*The spectrum* of the linear differential operator L(λ) consists of two parts: the essential spectrum and the isolated spectrum (point spectrum comprising the eigenvalues). Please see the Appendix for the definitions of the linear differential operators L(λ) and $L_{0}$. Are there any other spectrum in Ω={λ∈C:Reλ>−1} other than the essential spectrum and the isolated spectrum to the linear differential operator L(λ)? By assumption, the kernel functions *K* and *W* converge to zero exponentially fast as x→±∞. Therefore, the operator $[L(\lambda )-L_{0}]{\left(L_{0}\right)}^{-1}$ is a compact operator in $C_{0}(R)$. The residual spectrum does not exist in our model.

*The essential spectrum* is easy to calculate by following the original ideas of John Evans [14]. The complex number λ∈C belongs to the essential spectrum if and only if *λ* is a complex number such that the solutions of the differential equation

(17) $$\mu \psi^{\prime }+(\lambda +1)\psi =0,$$

is bounded on ℝ. The solution of this equation is given by $\psi (z)=C\exp(-\frac{\lambda +1}{\mu }z)$. This solution is bounded on ℝ if and only if λ=−1+ir, for some real number *r*. Since the subsequent sections show that linear deviations from stationary traveling wave front obey differential equations of the type (17), we find that the essential spectrum contains those values of *λ* with Reλ=−1<0 only, i.e., the essential spectrum does not threaten the stability of the traveling wave front.

It remains to investigate whether the isolated spectrum (the eigenvalues) threatens the stability, i.e., whether there are eigenvalues $\lambda_{0}$ with positive real part Reλ0>0, threatening the stability of the stationary front.

Define the Evans function E=E(λ) for the traveling wave front of Eq. (1) by

(18) $$E(\lambda )=\left[E_{1}(\lambda )+E_{2}(\lambda )\right]-1,$$

where $E_{1}=E_{1}(\lambda )$ and $E_{2}=E_{2}(\lambda )$ are also called Evans functions, defined by

(19) $$\begin{array}{rcl} E_{1}(\lambda ) & = & 1-\frac{\alpha }{\mu_{0}U^{\prime }(0)}\int_{0}^{\infty }\xi (c)\\ & & \times \left[\int_{-\infty }^{0}\exp\left((\lambda +1)\frac{c-\mu_{0}}{c\mu_{0}}x\right)\times \exp\left(\frac{\lambda }{c}x\right)K(x)dx\right]dc, \end{array}$$

(20) $$E_{2}(\lambda )=1-\frac{\beta }{\mu_{0}U^{\prime }(0)}\int_{0}^{\infty }\eta (\tau )e^{\tau }\left[\int_{-\infty }^{-\mu_{0}\tau }\exp\left(\frac{\lambda +1}{\mu_{0}}x\right)W(x)dx\right]d\tau ,$$

in the open domain Ω={λ∈C:Reλ>−1}. Here, the Evans function E=E(λ) is also called the stability index function and represents a sum of two single stability index functions $E_{1}=E_{1}(\lambda )$ and $E_{2}=E_{2}(\lambda )$, reflecting the contribution of transmission delay and constant delay, respectively.

*Properties of the Evans functions*: A complex number $\lambda_{0}$ is an eigenvalue of the eigenvalue problem L(λ)ψ=λψ (details to be given in the Appendix) if and only if $\lambda_{0}$ is a zero of the Evans function. Moreover,

$$\lim_{|\lambda |\to \infty }E_{1}(\lambda )=1,\;\lim_{|\lambda |\to \infty }E_{2}(\lambda )=1,\;\lim_{|\lambda |\to \infty }E(\lambda )=1.$$

### 2.4 The Numerical Simulations

To complement the analytical study, subsequent sections show numerical integrations of the model equation (1). To this end, if not stated otherwise, the spatial kernel functions are chosen to

$$K(x)=\frac{1}{2(s-r)}\left[s\exp\left(-|x|\right)-r\rho \exp\left(-\rho |x|\right)\right],\;W(x)=\frac{1}{2\sigma }e^{-|x|/\sigma },$$

with the real parameters s>r, ρ>1, σ>0. Equation (1) is integrated numerically in time by an Euler-forward method with time step △t=0.02, the spatial integral has been computed on a circular spatial grid of length L=60 and 600 intervals by the Monte Carlo-type VEGAS-algorithm [22] with 5000 random draws. If not stated differently, the initial values of the neural activity have been chosen to the analytical stationary front U(x−cT) perturbed by random values γ(x,t) taken from a uniform distribution γ(x,T)∈[−0.025,0.025] in the initial interval −12≤T≤0.

## 3 Results

This section presents the new findings on the effect of distributed delays in both standing wave front and traveling wave front. They extend previous results mentioned in the previous section.

### 3.1 The Standing Wave Front

*Derivation of an eigenvalue problem*. Recall that the standing wave front U=U(x) is a time independent solution of the nonlinear scalar integro-differential equation

$$\begin{array}{rcl} \frac{\partial u}{\partial t}+u & = & \alpha \int_{0}^{\infty }\xi (c)\left[\int_{R}K(x-y)H\left(u\left(y,t-\frac{1}{c}|x-y|\right)-\theta \right)dy\right]dc\\ & & +\beta \int_{0}^{\infty }\eta (\tau )\left[\int_{R}W(x-y)H\left(u(y,t-\tau )-\theta \right)dy\right]d\tau . \end{array}$$

To study the spectral stability of the stationary standing wave front, we linearize this equation about the front U=U(x) and we find that v(x,t)=u(x,t)−U(x) obeys the differential equation (neglecting higher order terms)

$$\begin{array}{rcl} & & \frac{\partial v}{\partial t}(x,t)+v(x,t)\\ & & \;=\alpha \int_{0}^{\infty }\xi (c)\left[\int_{R}K(x-y)s^{\prime }\left(U(y)-\theta \right)v\left(y,t-\frac{1}{c}|x-y|\right)dy\right]dc\\ & & \;+\beta \int_{0}^{\infty }\eta (\tau )\left[\int_{R}W(x-y)s^{\prime }\left(U(y)-\theta \right)v(y,t-\tau )dy\right]d\tau , \end{array}$$

where $s^{\prime }(U(y)-\theta )=\frac{\delta (y-x_{0})}{U^{\prime }(x_{0})}$, because $H(U(x)-\theta )=H(x-x_{0})$. Making a change of variable and using (7), we find the equation

(21) $$\begin{array}{rcl} & & \frac{\partial v}{\partial t}(x,t)+v(x,t)\\ & & \;=\frac{\alpha K(x-x_{0})}{\alpha K(0)+\beta W(0)}\int_{0}^{\infty }\xi (c)v\left(x_{0},t-\frac{1}{c}|x-x_{0}|\right)dc\\ & & \;+\frac{\beta W(x-x_{0})}{\alpha K(0)+\beta W(0)}\int_{0}^{\infty }\eta (\tau )v(x_{0},t-\tau )d\tau , \end{array}$$

where αK(0)+βW(0)>0. We understand that the contribution of the spatial interactions to the activity is maximum if $\left|K(x-x_{0})|/[\alpha K(0)+\beta W(0)\right]$ and $\left|W(x-x_{0})|/[\alpha K(0)+\beta W(0)\right]$ are large. For kernel functions with a maximum value at the origin, the largest contribution is expected at $x\approx x_{0}$, i.e., close to the threshold *θ*. Hence, one expects a large change of the activity close to the threshold *θ*. This is different from other spatial interactions, such as gamma-distributed kernel functions [27], where the strongest contribution occurs away from the threshold. 

A standing wave front is translation invariant. We may let $x_{0}=0$. Suppose that v(x,t)=ψ(x)exp(λt) is a solution of the above equation, where *λ* is a complex constant and ψ=ψ(z) is a bounded continuous function defined on ℝ. After canceling out exp(λt), we obtain the eigenvalue problem

$$\begin{array}{rcl} \left(\lambda +1)\psi (x\right) & = & [\frac{\alpha K(x)}{\alpha K(0)+\beta W(0)}\int_{0}^{\infty }\xi (c)\exp\left(-\frac{\lambda }{c}|x|\right)dc\\ & & +\frac{\beta W(x)}{\alpha K(0)+\beta W(0)}\int_{0}^{\infty }\eta (\tau )\exp(-\lambda \tau )d\tau ]\psi (0). \end{array}$$

The spectral stability of the stationary front (5) is determined by the eigenvalues of this eigenvalue problem. Letting x=0 in this equation and canceling out ψ(0) because it is not equal to zero, we get

(22) $$\lambda +1=\frac{\alpha K(0)}{\alpha K(0)+\beta W(0)}+\frac{\beta W(0)}{\alpha K(0)+\beta W(0)}\int_{0}^{\infty }\eta (\tau )\exp(-\lambda \tau )d\tau .$$

At first, we observe that the spectral stability of the standing wave front just depends on the spatial self-interactions K(0)/[αK(0)+βW(0)] and W(0)/[αK(0)+βW(0)], i.e., lateral interactions do not determine the stability of the stationary standing wave front. Moreover, finite transmission speeds do not affect the stability, but feedback delays may do. This result originates from the different nature of the delays: the transmission delay does not affect the dynamics at the threshold *θ* since it vanishes at zero distance from the threshold *θ* whereas the feedback delay is independent of the distance and affects the dynamics at all spatial locations.

*Spectral analysis*. Obviously, the neutral eigenvalue λ=0 is a trivial solution of this equation. To study the spectral stability of the standing wave front with respect to feedback delays, let us define the auxiliary function on the interval (−1,∞):

$$f(\lambda )\equiv \lambda +1-\frac{\alpha K(0)}{\alpha K(0)+\beta W(0)}-\frac{\beta W(0)}{\alpha K(0)+\beta W(0)}\int_{0}^{\infty }\eta (\tau )\exp(-\lambda \tau )d\tau .$$

For all kernel functions *K* and *W*, for all probability density functions *ξ* and *η*, *f* is an increasing of *λ*. There exists a unique solution $\lambda_{0}=0$ to the equation f(λ)=0. Moreover, $\lambda_{0}=0$ is a simple solution (that is, $\lambda_{0}=0$ is a simple eigenvalue). Therefore, the standing wave front is spectrally stable. Let us consider a very special case: absent intraarea connections (i.e., α=0) and focus on nonlocal feedback connections with a single feedback delay $\tau_{0}$ (i.e. $\eta (\tau )=\delta (\tau -\tau_{0})$, where $\tau_{0}>0$ is a positive constant). Then $\lambda +1=\exp(-\lambda \tau_{0})$. That is $(\lambda +1)\exp(\lambda \tau_{0})=1$. Given the positive constant $\tau_{0}>0$, it is very easy to see that there exists a unique solution $\lambda_{0}=0$ to $(\lambda +1)\exp(\lambda \tau_{0})=1$.

**Fig. 1:**
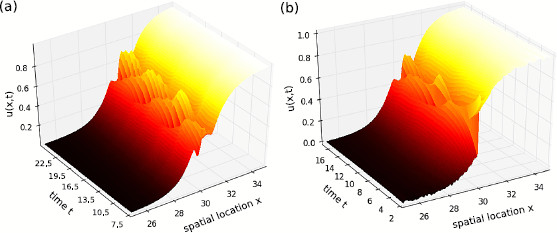
Propagating stationary wave front of Eq. (1) for delayed feedback with spatial feedback connections taken from kernel class (C) without intra-area connections. **a** Single delay with η(τ)=δ(τ−τ), $\tau_{0}=2.0$, **b** distributed delays $\eta (\tau )=\delta (\tau -\tau_{1})/2+\delta (\tau -\tau_{2})/2$ with $\tau_{1}=1.3$, $\tau_{2}=2.6$, i.e. E=1.95. In addition α=0, β=1.0, θ=0.5, W(x)=(exp(−|x|)−4.0exp(−10.0|x|))/1.2. Initial conditions are chosen to u(x,T)=0.0, 0≤x<L/2, u(x,T)=1.0, L/2≤x<L, $-\tau_{\max}\leq T\leq 0$ with the maximum delay $\tau_{\max}$ and spatial domain length L=60. The panels show a spatial and temporal extract of the full simulation result for visualization reasons

Moreover, Fig. 1 shows the space-time activity of oscillatory unstable standing front gained by a numerical simulation of Eq. (1) for a single delay (a) and a distribution of two delays (b). We observe oscillatory activity close to the threshold value *θ* consistent with the reasoning in Sect. 2.2. 

### 3.2 Traveling Wave Front

This subsection investigates analytically the uniqueness of the stationary travelling wave front and the spectral stability of the front. Numerical simulations validate the analytical findings.

#### 3.2.1 The Uniqueness

Taking a close look at the implicit equation (10), the question arises whether its solution, i.e., the wave speed $\mu_{0}$, is unique, or whether there are several possible traveling wave fronts with different wave speeds. Previous studies on propagating front in neural fields involving a single axonal finite transmission speed [11] have established the uniqueness of the traveling wave front. The present work extends these studies by considering distributed transmission speeds and distributed feedback delays. 

If the speed index functions $\phi_{1}(\mu )$, $\phi_{2}(\mu )$, and ϕ(μ) defined in (11), (12), and (10) are monotonic in *μ*, then the wave speed of the traveling wave front is unique. By using rigorous mathematical analysis (to keep the paper from too long, details are not to be given here), we find that 

• For all kernel functions (K,W) in classes (A) and (B), for all speed and delay distributions (ξ,η), for all $\mu \in (0,c_{0})$, there hold the following estimates:

(23) $$\frac{\partial \phi_{1}}{\partial \mu }(\mu )>0,\;\frac{\partial \phi_{2}}{\partial \mu }(\mu )>0.$$

• For all kernel functions (K,W) in class (C), for all speed and delay distributions (ξ,η), for all $\mu \in (0,c_{0})$, there hold the following estimates:

(24) $$\frac{\partial \phi_{1}}{\partial \mu }(\mu )<0\;\text{on }(0,\mu_{*}),\;\frac{\partial \phi_{1}}{\partial \mu }(\mu )>0\;\text{on }(\mu_{*},c_{0}),$$

(25) $$\frac{\partial \phi_{2}}{\partial \mu }(\mu )<0\;\text{on }(0,\mu_{**}),\;\frac{\partial \phi_{2}}{\partial \mu }(\mu )>0\;\text{on }(\mu_{**},c_{0}),$$

for two positive constants $\mu^{*}$ and $\mu^{**}$, where $0<\mu^{*}<c_{0}$ and $0<\mu^{**}<c_{0}$.

 In addition,

$$\lim_{\mu \to 0}\phi_{1}(\mu )=\lim_{\mu \to 0}\phi_{2}(\mu )=0$$

and hence $\phi_{1}(\mu^{*})<0$ and $\phi_{2}(\mu^{**})<0$. Since 0<2θ<α+β, condition (10) stipulates $\phi_{1}(\mu_{0})+\phi_{2}(\mu_{0})>0$. Consequently, it is necessary that $\frac{\partial \phi_{1}}{\partial \mu }(\mu )>0$ and $\frac{\partial \phi_{2}}{\partial \mu }(\mu )>0$ in at least a small neighborhood of the wave speed $\mu =\mu_{0}$.

Overall, there exists a unique wave speed and there is a unique traveling wave front to Eq. (1).

#### 3.2.2 Dependence on Transmission Speed and Feedback Delays

Now we investigate the change of the wave speed while changing the speed and delay distributions assuming the distributions (2). For simplicity, we choose $c_{2}/c_{1}=\tau_{2}/\tau_{1}=\kappa$, where *κ* is a fixed positive constant. Then (23), (24), and (25) and the definitions (11), (12) yield for general synaptic interactions *K* and *W* that

$$\begin{array}{rcl} & & \frac{\partial \phi }{\partial \mu_{0}}\frac{d\mu_{0}}{dc_{0}}+\frac{\partial \phi }{\partial c_{0}}=0,\\ & & \frac{d\mu_{0}}{dc_{0}}=-\frac{\partial \phi /\partial c_{0}}{\partial \phi /\partial \mu_{0}}>0, \end{array}$$

and for excitatory delayed feedback interaction, i.e., W(x)≥0,

$$\begin{array}{rcl} & & \frac{\partial \phi }{\partial \mu_{0}}\frac{d\mu_{0}}{d\tau_{0}}+\frac{\partial \phi }{\partial \tau_{0}}=0,\\ & & \frac{d\mu_{0}}{d\tau_{0}}=-\frac{\partial \phi /\partial \tau_{0}}{\partial \phi /\partial \mu_{0}}<0. \end{array}$$

**Fig. 2:**
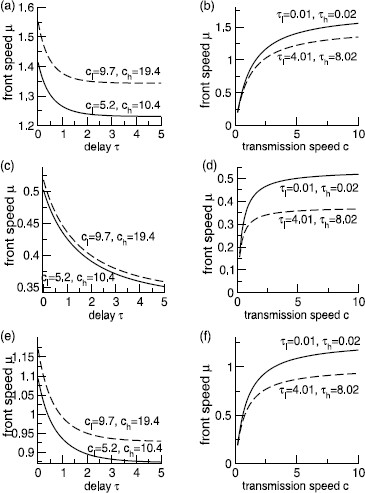
Wave speeds for different transmission speed and feedback delay distributions in class (A), (B) and (C) of the spatial kernel function *K* with an excitatory feedback W≥0. The transmission speed and feedback delay distributions are taken from Eqs. (2) with $c_{1}=2c_{0}$ and $\tau_{1}=2\tau_{0}$. *Panels***a** and **b** give the speeds for class (A), *panels***c** and **d** for class (B) and *panels***e** and **f** for class (C). The delays *τ* in **a**, **c** and **e** are identical to the lowest delays $\tau_{0}$, the transmission speeds *c* in **b**, **d** and **f** are identical to the lowest transmission speed $c_{0}$ in the distribution. Other kernel parameters are taken from Fig. 3

Figure 2 shows the wave speed $\mu_{0}$ given implicitly by (10) with respect to different transmission speed distributions and feedback delay distributions corresponding to (2) for the kernel functions in the three classes (A), (B), and (C). 

#### 3.2.3 Stability Analysis

*Analysis of the eigenvalues of the eigenvalue problem*. The introduction of the speed index functions and the stability index functions given in Sect. 2.3.3 reveals novel relationship between the uniqueness of the wave speed and the stability of the traveling wave front. Comparing the stability index functions given in Eqs. (19), (20) and the speed index functions in Eqs. (11), (12), we can obtain the following results. 

(I) For intracortical interactions only (i.e., α>0, β=0, and 0<2θ<α), the speed index function $\phi_{1}$ from Eq. (11) and the stability index function E(λ) from (18) are related by

(26) $$E(\lambda )=E_{1}(\lambda )=1-\frac{1}{\phi_{1}(\mu_{0})}\phi_{1}\left(\frac{\mu_{0}}{\lambda +1}\right).$$

Let E(λ)=0, then

(27) $$\phi_{1}(\mu_{0})=\phi_{1}(\gamma \mu_{0}),\;\text{where }\gamma =\frac{1}{\lambda +1}\in C.$$

For the eigenvalue $\lambda_{0}=0$, we find that γ=1, and hence (27) holds. This eigenvalue reflects the translation invariance of the stationary traveling wave front. To study the spectral stability of the front, the question arises whether there is another complex eigenvalue $\lambda_{0}$ with positive real part Reλ0>0 for which (27) holds. Since the kernel function *K* is real, it follows that the eigenvalue $\lambda_{0}$ (if it exists) is real and γ<1 if $\lambda_{0}>0$. Then Eq. (27) breaks down to the question whether there is another solution $\gamma \mu_{0}<\mu_{0}$ or, equivalently, whether $\phi_{1}(\mu )$ is monotonic in $\left(0,c_{0}\right)$. This question has already been answered in the context of the uniqueness of the wave speed in Sect. 2.3.2. Since $\mu_{0}$ is the unique solution of Eq. (10), Eq. (27) has a unique solution γ=1 so $\lambda_{0}=0$ is the only eigenvalue.

(II) Now let us consider feedback connections only (i.e., α=0, β>0, and 0<2θ<β). The stability index function (18) reads

$$E(\lambda )=1-\frac{\beta }{\mu_{0}U^{\prime }(0)}\int_{0}^{\infty }\eta (\tau )e^{\tau }\left[\int_{-\infty }^{-\mu_{0}\tau }\exp\left(\frac{\lambda +1}{\mu_{0}}x\right)W(x)dx\right]d\tau .$$

Then (10), (13), (14), and (15) yield

(28) $$\begin{array}{rcl} & & \int_{0}^{\infty }\eta (\tau )e^{\tau }\left[\int_{-\infty }^{-\mu_{0}\tau }\exp\left(\frac{\lambda +1}{\mu_{0}}x\right)W(x)dx\right]d\tau \\ & & \;=\int_{0}^{\infty }\eta (\tau )e^{\tau }\left[\int_{-\infty }^{-\mu_{0}\tau }\exp\left(\frac{x}{\mu_{0}}\right)W(x)dx\right]d\tau . \end{array}$$

Since instabilities imply Reλ0>0, $|\exp(\lambda_{0}x/\mu_{0})|<1$, the left-hand side of (28) is smaller than its right-hand side, and hence (28) holds true only for $\lambda_{0}=0$, i.e., the front is spectrally stable.

(III) For both intracortical and feedback connections (i.e., α>0, β>0, and 0<2θ<α+β), the stability index function reads

$$E(\lambda )=1-\frac{1}{\phi_{1}(\mu_{0})+\phi_{22}(\mu_{0})}\left[\phi_{1}\left(\frac{\mu_{0}}{\lambda +1}\right)+\phi_{22}\left(\frac{\mu_{0}}{\lambda +1}\right)\right].$$

Similar to cases (I) and (II), we obtain the relationship

(29) $$\phi_{1}(\mu_{0})+\phi_{22}(\mu_{0})=\phi_{1}(\gamma \mu_{0})+\phi_{22}(\gamma \mu_{0}).$$

By virtue of the monotonicity of the speed index function $\phi_{1}(\mu )+\phi_{22}(\mu )$, γ=1 is the only solution of (29), i.e., $\lambda_{0}=0$ is the only eigenvalue and the front is spectrally stable.

Now let us consider distributed feedback delays, the stability index function (18) and Eqs. (10), (15) yield

(30) $$\begin{array}{rcl} & & \alpha \int_{0}^{\infty }\xi (c)\left[\int_{-\infty }^{0}\exp\left(\frac{c-\mu_{0}}{c\mu_{0}}x\right)K(x)dx\right]dc\\ & & \;+\beta \int_{0}^{\infty }\eta (\tau )e^{\tau }\left[\int_{-\infty }^{-\mu_{0}\tau }\exp\left(\frac{x}{\mu_{0}}\right)W(x)dx\right]d\tau \\ & & \;=\alpha \int_{0}^{\infty }\xi (c)\left[\int_{-\infty }^{0}\exp\left(\frac{c-\mu_{0}}{c\mu_{0}}x\right)\exp\left(\frac{\lambda }{\mu_{0}}x\right)K(x)dx\right]dc\\ & & \;+\beta \int_{0}^{\infty }\eta (\tau )e^{\tau }\left[\int_{-\infty }^{-\mu_{0}\tau }\exp\left(\frac{\lambda +1}{\mu_{0}}x\right)W(x)dx\right]d\tau . \end{array}$$

Since $|\exp(\lambda x/\mu_{0})|<1$ for all *λ* with Reλ>0, the same reasoning as in (I) and (II) applies and the right side of Eq. (30) is smaller than its left side. Hence, the only solution of Eq. (30) is $\lambda_{0}=0$. Moreover, the derivative of the Evans function at λ=0 is positive, that is $E^{\prime }(0)>0$. In another words, the neutral eigenvalue λ=0 is a simple eigenvalue.

On the other hand, note that

$$\lim_{|\lambda |\to \infty }E(\lambda )=1,$$

and

$$\max_{\lambda \in iR}\left|E(\lambda )\right|=1.$$

**Fig. 3:**
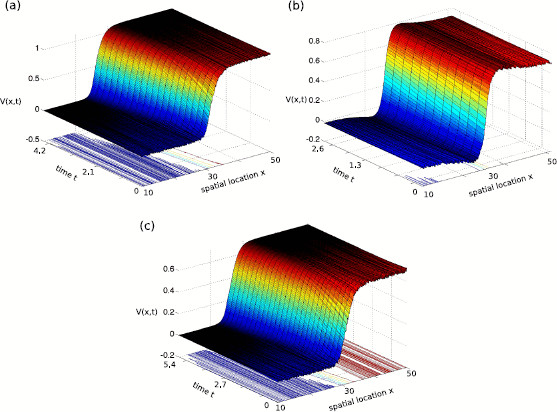
Propagating stationary wave front of Eq. (1) for three classes of synaptic interactions and distributed speeds and delays. The kernel functions *K* and *W* are chosen from the class (A) (*panel***a**), (B) (*panel***b**) and (C) (*panel***c**). In **a**r=0, **b**r=0.4, ρ=0.2 and **c**r=0.4, ρ=3.0. Other parameters are s=1, α=1.0, β=0.1, σ=1.0, θ=0.2, and the speed and delay distributions are $\xi (c)=[\delta (c-c_{0})+\delta (c-c_{1})]/2$, $\eta (\tau )=[\delta (\tau -\tau_{0})+\delta (\tau -\tau_{1})]/2$ with $c_{0}=5.0$, $c_{1}=10.0$, $\tau_{0}=0.1$ and $\tau_{1}=0.2$

We write λ=x+iy and $E(\lambda )=E_{\mathrm{real}}(\lambda )+iE_{\mathrm{imag}}(\lambda )$. Note that both the real part $E_{\mathrm{real}}(\lambda )$ and the imaginary part $E_{\mathrm{imag}}(\lambda )$ of the Evans function E(λ) are real harmonic functions of *x* and *y*. Hence, they satisfy the mean value formula; see Evans [14-17]. As a result, E(λ) also satisfies the mean value formula. Thus, |E(λ)| cannot attain a local maximum inside any open domain of ℂ. By using a strong maximum principle of |E(λ)| in *Ω*, we find that 0<|E(λ)|<1, for all λ∈C with Reλ>0. The spectral stability follows immediately. To illustrate this finding, Fig. 3 shows numerical simulations of Eq. (1) and we observe stationary propagating front for kernel functions in the three classes (A), (B), and (C). 

The simulations consider both distributed axonal transmission speeds and distributed feedback delays. We observe that starting from a noise-perturbed stationary front, the activity approaches the smooth stationary propagating front after some time, i.e., the front is also exponentially stable in the presence of feedback delay. This numerical result for nonvanishing feedback connections complements the analytical result for vanishing feedback delay above.

## 4 Discussion

### 4.1 Stability of the Standing Wave Front

The results in Sect. 3.1 show that transmission delays do not destabilize standing wave front. In contrast, nonlocal feedback delays may destabilize standing wave front for certain delays resulting to oscillatory activity. The corresponding analytical conditions for a single delay hold for feedback connections in class (C), i.e., for local inhibition–lateral excitation interactions. Since such synaptic couplings as well as feedback delays are omnipresent in the brain, oscillatory standing wave front may occur frequently in real neural structures. For distributed delays, such instability may be possible, but no analytical method is known up to today proving oscillatory instability. Nevertheless, numerical simulations of standing wave front subject to distributed feedback delays reveal oscillatory instability as well.

### 4.2 Wave Speed of the Traveling Wave Front

Section 3.2 shows that the speed index function is monotonically increasing, i.e., $\frac{\partial \phi }{\partial \mu }>0$ on the interval where the wave speed exist. Consequently, the wave speed is unique. Moreover, the same subsection shows analytically that the increase of the transmission speed, i.e., the decrease of the transmission delay, increases the wave speed. Similarly, the increase of the feedback delays decreases the wave speed. Summarizing these results, increasing delays slows down the wave speed of the traveling wave front.

### 4.3 Stability of the Travelling Wave Front

The results in Sect. 3.2 indicate that traveling wave front is spectrally stable in the absence of delayed feedback due to the uniqueness of the wave speed. To our best knowledge, this relationship between the uniqueness and the stability has not been found before, although claimed in previous studies [50,51]. 

Focusing on the effect of delayed feedback, the stability analysis in Sect. 3.2.3 reveals the stability of traveling wave front for distributed delays. To support the analytical results, numerical simulations were performed showing stable traveling wave front, cf. Fig. 3. Additional extensive numerical studies (not shown) on the effect of distributed feedback delays have found stable traveling wave front only.

Summarizing the latter results, distributed transmission delays do not destabilize traveling wave front, but feedback delays may induce oscillatory instability.

### 4.4 Summary and Outlook

We find that the standing wave front and the stationary traveling wave front involving distributed transmission speeds exhibit a unique stable traveling wave front $u(x,t)=U(x+\mu_{0}t)$ to Eq. (1) given any pair of synaptic couplings (*K*, *W*), probability density functions (*ξ*, *η*), synaptic rate constants (*α*, *β*), any threshold *θ* and if assumptions (3) hold. In addition, the standing wave front and the stationary traveling wave front are spectrally stable in the presence of small external perturbations due to the found relationship between uniqueness and stability. In contrast, the additional presence of nonlocal feedback delays may render the stationary front instability. We find that the delay-induced loss of stability is oscillatory. This is valid for the present model involving a single synaptic time scale. It is worth mentioning, however, that neural fields involving multiple synaptic time scales are sensitive to transmission delays [33]. 

Future work may further investigate the relation of uniqueness and stability in other wave phenomena, such as traveling pulse solutions or global waves. Moreover, it is interesting to further study the impact of nonlocal delayed feedback on these wave phenomena. These studies may permit deeper insight into the role of delayed feedback loops which are omnipresent in neural systems.

## Appendix

Let $U=U_{\mathrm{front}}(z)$ represent the traveling wave front of the nonlinear scalar integro-differential equation (1).

**Definition 1** (Eigenvalue problems, eigenvalues, and eigenfunctions)

Let λ∈C and Reλ>−1. Define a linear differential operator by

$$L_{0}\psi =-\mu \psi^{\prime }-\psi .$$

Define a family of linear differential operators L(λ) by using the eigenvalue problem

(31) L(λ)ψ=λψ.

The eigenvalue problem is given explicitly by

$$\begin{array}{rcl} & & \mu \psi^{\prime }(z)+(\lambda +1)\psi (z)\\ & & \;=\frac{\alpha }{U^{\prime }(0)}\left\{\int_{0}^{\infty }\xi (c)\left[\frac{c}{c+s(z)\mu }K\left(\frac{cz}{c+s(z)\mu }\right)\right]\exp\left[-\frac{\lambda |z|}{c+s(z)\mu }\right]dc\right\}\psi (0)\\ & & \;+\frac{\beta }{U^{\prime }(0)}\left\{\int_{0}^{\infty }\eta (\tau )\left[W(z-\mu \tau )\right]\exp(-\lambda \tau )d\tau \right\}\psi (0). \end{array}$$

If there exists a complex number $\lambda_{0}$ and there exists a nontrivial bounded continuously differentiable function $\psi_{0}$ defined on ℝ, such that $L(\lambda_{0})\psi_{0}=\lambda_{0}\psi_{0}$, then $\lambda_{0}$ is called an eigenvalue and $\psi_{0}$ is called an eigenfunction of the eigenvalue problem.

**Definition 2** (The spectral stability)

(1) If max{Reλ:λ≠0,λ∈σ(L(λ))}≤−C0 and if $\lambda_{0}=0$ is a simple eigenvalue of the eigenvalue problem L(λ)ψ=λψ, then we say that the traveling wave front is spectrally stable, where σ(L(λ)) represents the spectrum of the operator L(λ), $C_{0}>0$ a positive constant.

(2) If there exists an eigenvalue with positive real part to the eigenvalue problem L(λ)ψ=λψ, then we say that the traveling wave front is exponentially unstable.

(3) If max{Reλ:λ≠0,λ∈σ(L(λ))}<0, that is, there exists no nonzero eigenvalue to the eigenvalue problem L(λ)ψ=λψ in the right half plane {λ∈C:Reλ>0}, but $\lambda_{0}=0$ is not a simple eigenvalue of the eigenvalue problem L(λ)ψ=λψ, then we say that the traveling wave front is algebraically unstable.

Therefore, we have finished the proof of the spectral stability.

## Competing Interests

The authors declare that no competing interests exist.

## Authors’ Contributions

AH carried out the numerical simulations, AH and LZ designed the study and performed the analytical computations. Both authors read and approved the final manuscript.
